# Higher Ventricular-Arterial Coupling Derived from Three-Dimensional Echocardiography Is Associated with a Worse Clinical Outcome in Systemic Sclerosis

**DOI:** 10.3390/ph14070646

**Published:** 2021-07-05

**Authors:** Francesco Tona, Elisabetta Zanatta, Roberta Montisci, Denisa Muraru, Elena Beccegato, Elena De Zorzi, Francesco Benvenuti, Giovanni Civieri, Franco Cozzi, Sabino Iliceto, Andrea Doria

**Affiliations:** 1 Department of Cardiac, Thoracic, Vascular Sciences and Public Health, 35128 Padova, Italy; denisa.muraru@unipd.it (D.M.); elena.beccegato@unipd.it (E.B.); giovanni.civieri@yahoo.it (G.C.); sabino.iliceto@unipd.it (S.I.); 2 Department of Medicine, Padova University Hospital, 35128 Padova, Italy; elisabettazanatta86@gmail.com (E.Z.); elena.dezorzi@gmail.com (E.D.Z.); fran.benvenuti@gmail.com (F.B.); franco.cozzi@unipd.it (F.C.); andrea.doria@unipd.it (A.D.); 3 Clinical Cardiology, AOU Cagliari, Department of Medical Science and Public Health, University of Cagliari, 09042 Cagliari, Italy; rmontisc@gmail.com

**Keywords:** heart failure, 3D-echocardiography, ventricular function, outcome, systemic sclerosis, ventricular-arterial coupling

## Abstract

Primary myocardial involvement is common in systemic sclerosis (SSc). Ventricular-arterial coupling (VAC) reflecting the interplay between ventricular performance and arterial load, is a key determinant of cardiovascular (CV) performance. We aimed to investigate VAC, VAC-derived indices, and the potential association between altered VAC and survival free from death/hospitalization for major adverse CV events (MACE) in scleroderma. Only SSc patients without any anamnestic and echocardiographic evidence of primary myocardial involvement who underwent three-dimensional echocardiography (3DE) were included in this cross-sectional study and compared with healthy matched controls. 3DE was used for noninvasive measurements of end-systolic elastance (Ees), arterial elastance (Ea), VAC (Ea/Ees) and end-diastolic elastance (Eed); the occurrence of death/hospitalization for MACE was recorded during follow-up. Sixty-five SSc patients (54 female; aged 56 ± 14 years) were included. Ees (*p* = 0.04), Ea (*p* = 0.04) and Eed (*p* = 0.01) were higher in patients vs. controls. Thus, VAC was similar in both groups. Ees was lower and VAC was higher in patients with diffuse cutaneous form (dcSSc) vs. patients with limited form (lcSSc) (*p* = 0.001 and *p* = 0.02, respectively). Over a median follow-up of 4 years, four patients died for heart failure and 34 were hospitalized for CV events. In patients with VAC > 0.63 the risk of MACE was higher (HR 2.5; 95% CI 1.13–5.7; *p* = 0.01) and survival free from death/hospitalization was lower (*p* = 0.005) than in those with VAC < 0.63. Our study suggests that VAC may be impaired in SSc patients without signs and symptoms of primary myocardial involvement. Moreover, VAC appears to have a prognostic role in SSc.

## 1. Introduction

Systemic sclerosis (SSc) is a chronic systemic autoimmune disease characterized by widespread vascular lesions and fibrosis of skin and internal organs [1]. Although often clinically silent [2,3], primary cardiac involvement is one of the main causes of death in SSc [4,5]. Thus, a yearly transthoracic echocardiography is recommended in patients with SSc to assess systolic pulmonary artery pressure as well as diastolic and systolic function of the left ventricle (LV) [6]. In this regard, some measurements such as end-diastolic diameter, fractional shortening, or LV ejection fraction (LVEF) are routinely used in clinical practice. However, these indices are load-dependent and do not systematically reflect the contractile state of the myocardium [7]. The interplay between cardiac function and arterial system—commonly defined as ventricular-arterial coupling (VAC)—is a major determinant of ventricular performance as it reflects global cardiovascular (CV) efficiency [8], and can be mathematically expressed as the ratio between arterial elastance (Ea) and end-systolic elastance (Ees) of the LV. VAC has been recently recognized as a key determinant of cardiovascular performance, and in fact, ventricular-arterial uncoupling which occurs in various clinical conditions, may predict morbidity and mortality [9,10,11].

The advantages of three-dimensional echocardiography (3DE) vs. 2-dimensional echocardiography (2DE) lie in its better accuracy, precision, and reproducibility for volume measurements [12], and consequently for VAC assessment [13].

We aimed to investigate VAC by 3DE in SSc patients, as well as potential differences in VAC values and VAC-derived indices by comparing patients with a limited and diffuse cutaneous form of SSc (lcSSc and dcSSc, respectively). Moreover, we set out to evaluate a potential association between altered VAC and survival-free from major adverse cardiovascular events (MACEs) in SSc.

## 2. Results

### 2.1. Echocardiography and Pressure-Volume Curve Parameters in SSc Patients and Controls

Baseline characteristics of the 65 patients enrolled in the study are shown in Table 1. LV diastolic dimension, wall thickness, and mass index were comparable in patients and in controls. Regional contractility was normal in all patients and controls. Left ventricular end-systolic volume (LVESV), LV end-diastolic volume (LVEDV), stroke volume (SV), and LVEF were similar in both groups. Systolic and diastolic blood pressure were comparable in patients and controls. E/e’ was higher in patients vs. controls (10.02 ± 4.3 vs. 6.5 ± 2.2, *p* < 0.0001). Ees and Ea were higher in patients vs. controls (3.95 ± 1.8 vs. 2.99 ± 0.7 mmHg/mL, *p* = 0.002; 2.28 ± 0.11 vs. 1.73 ± 0.07 mmHg/mL, *p* = 0.001, respectively), whereas VAC was comparable in both groups (0.60 ± 0.1 vs. 0.62 ± 0.2, *p* = 0.59). Diastolic elastance (Eed) was higher in patients (0.23 ± 0.01 vs. 0.16 ± 0.03 mmHg/mL, *p* = 0.001). Stroke work (SW), potential energy (PE), pressure-voulme area (PVA) and LV efficiency indicating mechanical energy exerted by the left ventricle were similar in both groups.

### 2.2. Echocardiography and Pressure-Volume Curve Parameters According to VAC Value

Patients in the higher VAC group (>0.63) had significantly higher LVESV (*p* < 0.0001) with reduced LVEF (*p* < 0.0001) than those with lower VAC (≤0.63). Ees was lower in patients with VAC > 0.63 (*p* < 0.0001) whereas Ea was similar in both groups (Table 1). Disease duration was longer (*p* = 0.03) and the prevalence of diffuse cutaneous SSc (dcSSc) was higher (*p* = 0.03) in patients with VAC > 0.63. Ongoing medications were comparable between the two groups.

### 2.3. Echocardiography and Pressure-Volume Curve Parameters in dcSSc and lcSSc Patients

**Figure 1:**
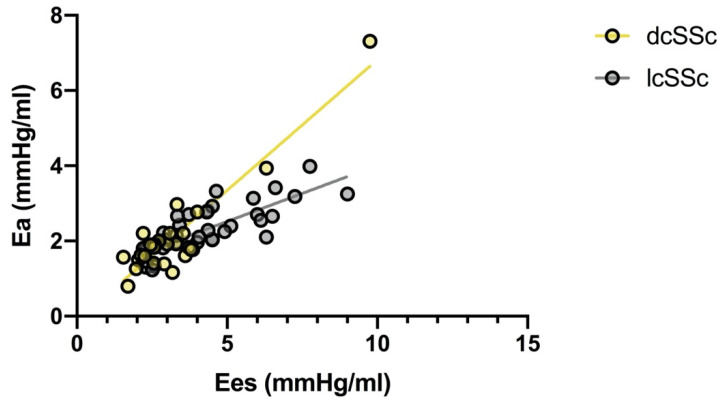
Scatterplot of the relationship between Ea and Ees in patients with lcSSc and patients with dcSSc. Ees correlates with Ea both in lcSSc (ρ = 0.779, *p* < 0.0001) and, albeit more weakly, in dcSSc (ρ = 0.599, *p* = 0.002).

Table 2 shows the differences between patients with dcSSc vs. lcSSc. In particular, LVEDV (*p* = 0.004), LVESV (*p* = 0.001), and SV (*p* = 0.03) were higher in dcSSc patients and LVEF was lower, albeit within the normal range (*p* = 0.01). Ea (*p* = 0.01) and Ees (*p* = 0.001) were lower in dcSSc patients. VAC was significantly higher in dcSSc patients (*p* = 0.02). PE was higher in dcSSc (*p* = 0.01) and LV efficiency was lower (*p* = 0.02) (Table 2). Ees correlated with Ea (ρ = 0.851, *p* < 0.0001). However, in dcSSc the correlation line is shifted upward and to the left. For the same Ea value, patients with dcSSc presented a lower Ees, indicative of inadequate contractility (Figure 1).

### 2.4. Correlations of Pressure-Volume Curve Parameters

Unlike Ees and VAC (ρ = −0.456, *p* < 0.0001 and ρ = 0.336, *p* = 0.008, respectively), Ea did not correlate with time elapsed from SSc diagnosis (ρ = 0.035, *p* = 0.78). Ea positively correlated with Eed (ρ = 0.857, *p* < 0.0001) and systolic pulmonary arterial pressure (ρ = 0.401, *p* = 0.002), and inversely with TAPSE (ρ = −0.434, *p* = 0.007). Ees positively correlated with Eed (ρ = 0.811, *p* < 0.0001). Eed inversely correlated with TAPSE (ρ = −0.447, *p* = 0.001).

### 2.5. Association between VAC and Other Clinical Variables

At univariate linear regression analysis, diagnosis of dcSSc (*p* = 0.009), therapy with prostanoid (*p* = 0.03), disease duration (*p* = 0.01) and age at diagnosis (*p* = 0.02) were determinants of VAC. To further investigate the potential factors involved in VAC alterations, we performed a multivariable linear regression (stepwise) including significant factors at univariate linear regression analysis which revealed that only diagnosis of dcSSc had an independent influence on VAC (Table 3).

### 2.6. Factors Associated with VAC > 0.63

In univariable logistic regression VAC > 0.63 was associated with time elapsed from diagnosis (*p* = 0.01), age at SSc onset (*p* = 0.04), diagnosis of dcSSc (*p* = 0.03), and LVESV (*p* = 0.002). In multivariable logistic regression, adjusted for age and sex, VAC > 0.63 was associated with LVESV (OR 1.076; 95% CI 1.012–1.144; *p* = 0.02) and time elapsed from diagnosis (OR 1.057; 95% CI 1.008–1.127; *p* = 0.04).

### 2.7. Major Adverse Cardiac Events

During a 4-year median follow-up (IQR, 2–10 years), 38 patients (58.5%) developed major adverse cardiac events (MACEs). Four patients (6%) died from heart failure, 16 (24%) were hospitalized for heart failure and 18 (28%) for angina (n = 12, 67%; nine without coronary epicardial stenosis and three with epicardial coronary stenosis), or myocardial infarction (n = 6, 33%). Twelve out of 16 (75%) of the heart failure episodes were with low ejection fraction (HFrEF). No heart failure episode was of right-sided origin. There were non-cardiovascular death or events during the follow-up period.

**Figure 2:**
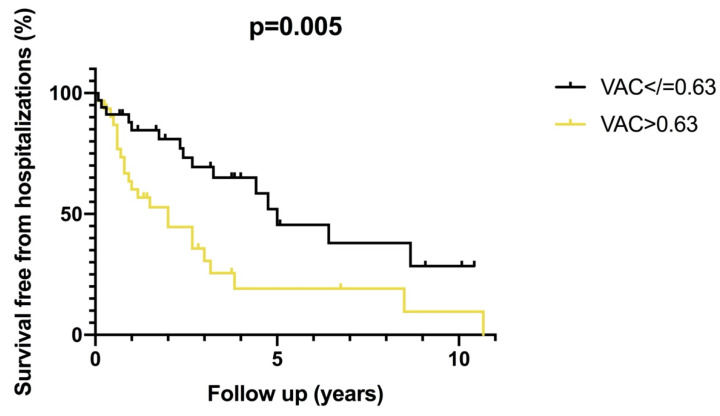
Kaplan Meier estimate of survival free from hospitalizations of patients with VAC ≤ 0.63 (in black), and patients with VAC > 0.63 (in yellow).

Differences between patients with and without MACEs are shown in Table 4. Time from SSc diagnosis was longer and LVEF was lower in patients with MACEs (*p* = 0.03 and *p* = 0.01, respectively). LVESV tended to be greater in patients with MACEs (*p* = 0.06). Ea was similar in patients with and without MACEs (*p* = 0.52). Ees was lower (*p* = 0.01) and VAC was higher (*p* = 0.008) in patients with MACEs. LV efficiency was lower in patients with MACEs (*p* = 0.01). VAC was >0.63 in 23/38 (60%) patients with MACEs and in 8/27 (29%) patients without MACEs (*p* = 0.01). Figure 2 shows the cumulative survival free from MACEs according to VAC value.

### 2.8. Risk Factors for MACEs in the Study Cohort

**Figure 3:**
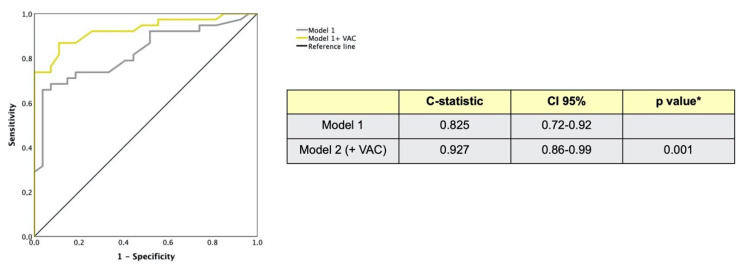
Receiving operating curves in model 1 and model 2 (including VAC > 0.63) for MACEs. C-statistic improves adding VAC > 0.63 in the multivariable model * *p* value is derived from comparison of model 1 to model 1 plus VAC > 0.63 (model 2).

In univariable Cox regression analysis, MACEs were associated with VAC > 0.63 (*p* = 0.008), LVEF < 62% (*p* = 0.02), LV efficiency < 76% (*p* = 0.02) and disease duration (*p* = 0.01). In the final multivariable regression model, also adjusted for age, sex, pulmonary hypertension, dcSSc and interstitial lung disease, VAC > 0.63 was independently associated with MACEs (HR 2.5; 95% CI 1.13–5.7; *p* = 0.01) (Table 5). The C statistic for multivariable model increased from 0.82 to 0.92 when adding VAC > 0.63 (*p* = 0.001) (Figure 3).

### 2.9. Incremental Value of VAC for Predicting Adverse Cardiac Events

**Figure 4:**
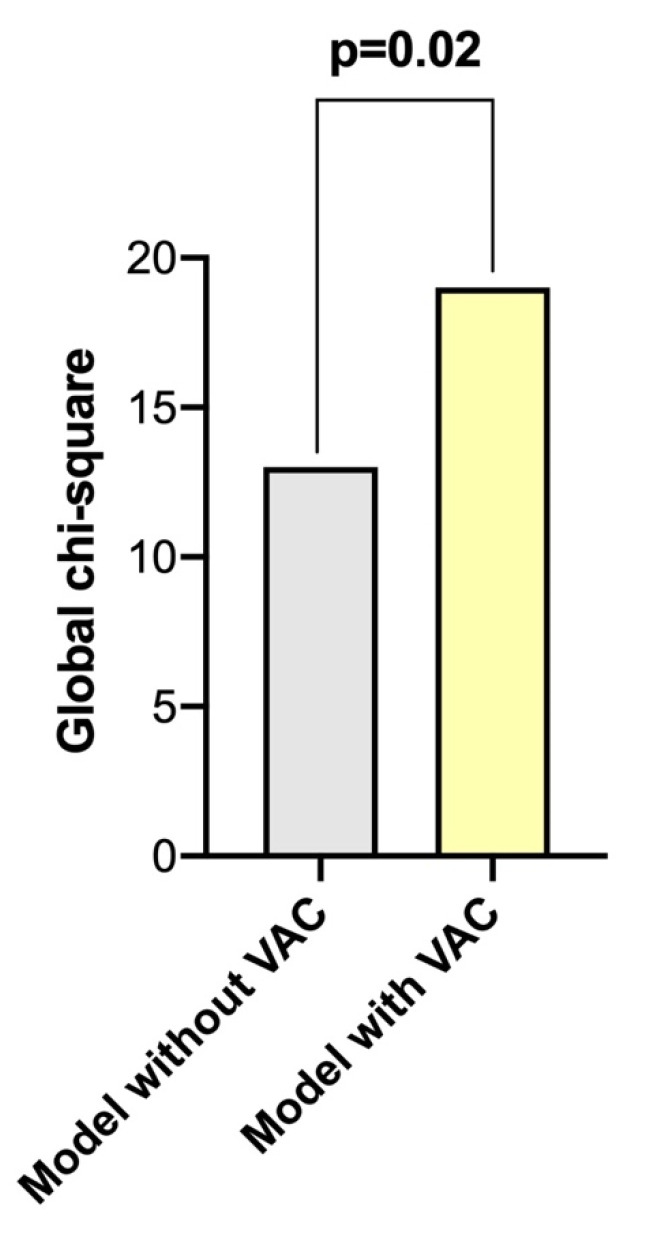
Incremental prognostic value of VAC > 0.63 when added to LVEF, LV efficiency, disease duration, age, sex, pulmonary hypertension, dcSSc and interstitial lung disease (Model 1).

To assess the incremental prognostic value of VAC, global chi-square scores were calculated (Figure 4). The addition of VAC > 0.63 (global chi-square: 13.1) significantly increased the global chi-square score (19.2; *p* = 0.02).

### 2.10. Intra and Interobserver Reproducibility of VAC by 3D

Intraobserver reproducibility was high (r = 0.98, SEE = 0.12); the mean difference was −0.02 and the upper and lower limits of agreement between the measurements were +0.14 (95% CI, +0.08 to +0.2) and −0.19 (95% CI, −0.26 to −0.13), respectively; intraclass correlation coefficient was 0.986. Interobserver reproducibility was also high (r = 0.96, SEE =0.18); the mean difference was 0.01 and the upper and lower limits of agreement between the 2 measurements were +0.36 (95% CI, +0.26 to +0.45) and −0.33 (95% CI, −0.43 to −0.23), respectively; intraclass correlation coefficient was 0.966.

### 2.11. Ventricular-Arterial Coupling by 2D and 3D Echo Modalities

Table 6 presents the comparison between 2D and 3D parameters. Although Ea and VAC were similar between 2D and 3D echocardiography, Ees was lower by 3D echocardiography.

**Figure 5:**
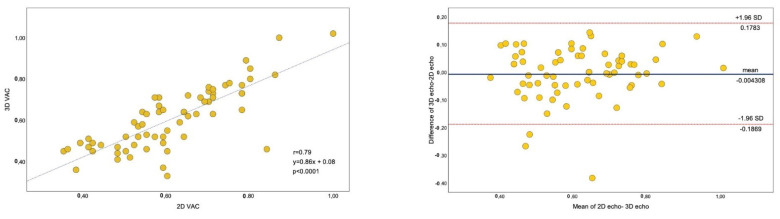
Linear regression (**left**) and Bland-Altman analysis (**right**) for VAC between 2D and 3D-echocardiography. Scattergram (**left** panel) showing the correrelation plot between VAC obtained by 2D- and 3D echocardiography. Plot of the difference (**right** panel) between the VAC measurements against their mean is shown. Medial line represents bias while the upper and lower red dotted lines the levels of agreement Dotted lines represent boundaries of means ± 2 SD, from −1.96 to +1.96. Relative mean error was calculated by the ratio of absolute difference of two values over their average.

Figure 5 presents a linear regression plot (left panel) and Bland–Altman analysis (right panel) for VAC computed by 2D- and 3D-echocardiography.

## 3. Discussion

Standard transthoracic measurements derived from echocardiography such as end-diastolic diameter, fractional shortening, or LVEF are routinely used in clinical practice. However, these indices are load-dependent and do not systematically reflect the contractile state of the myocardium.

Our main findings indicate that: (1) VAC by 3DE may be significantly higher in dcSSc patients than in lcSSc patients, despite normal LVEF and worsen in relation to disease duration; (2) VAC by 3DE may predict major cardiovascular events in SSc.

As LV and arterial system are anatomically continuous, their interaction is a crucial determinant of cardiovascular function [14,15]. Notably, and to the best of our knowledge, this is the first study to assess LV pressure-volume relationship and VAC by 3DE in SSc. 3DE allows for a more precise evaluation of LV volumes than 2D echocardiography [12] and this is paramount for a correct assessment of VAC. Comparison with two-dimensional measurements was beyond the scope of our study. Nevertheless, in our 65 patients we found a Pearson correlation r = 0.87 between 2D and 3D echocardiography (*p* = 0.0001) (data not shown).

In our study, the traditional indices of the LV (i.e., LVEDV, LVESV and LVEF) in SSc patients were similar to that observed in controls and SV tended to be lower (*p* = 0.07) in the former. However, Ea was higher and Ees was significantly higher in SSc patients. Thus, VAC was similar in SSc patients and controls. Eed was higher in patients, indicating high filling pressure. This hemodynamic arrangement is peculiar to heart failure with preserved ejection fraction (HF*p*EF) [11,14], one of the typical and prognostically negative clinical manifestations of cardiac involvement in SSc. We corroborated previous reports indicating high frequencies of impaired diastolic function in SSc. A recent study conducted on a large and unselected SSc cohort showed more frequent and severe diastolic dysfunction (2016 guidelines definition) during the disease course and a high impact on mortality in SSc [16].

Many studies have reported a low prevalence of systolic dysfunction in SSc patients [3,17,18]. However, we hypothesize that conventional echocardiography may cause LV systolic dysfunction to be underestimated. Although we found no differences in the diastolic function between dcSSc and lcSSc, as previously reported [16], there appears to be significant hemodynamic differences between the two main subgroup of SSc patients with different cutaneous form. In fact, our findings point to a predominant intrinsic LV systolic dysfunction in dcSSc and LV inability to compensate higher afterload, rather than important differences in load. The higher afterload in SSc may be attributable to increased arterial stiffness from deposition of collagen and other matrix components [19]. This is supported by the higher Ea value found in our study and correlates with a worse prognosis. In fact, in the recent consensus on the role of VAC [11] the Authors highlighted that extracellular matrix and cytoskeleton regulation processes are biochemical pathways that concomitantly affect cardiac and arterial structure and function through replacement or reactive fibrosis, which typically occurs in in SSc patients. The same Authors note that the measurement of VAC may be useful for not only SSc patients but also for patients with other cardiovascular diseases [11].

The inability of the contractile function of the myocardium to adapt to the afterload is evident from our results, mostly in patients with dcSSc. Impaired contractility and ventricular-arterial uncoupling may stem from coronary microvascular dysfunction and remodeling [20]. Moreover, VAC may be associated with future risk of coronary events due to microvascular dysfunction rather than coronary epicardial atherosclerotic stenosis. Endothelial-derived nitric oxide, oxidative stress and cytokines are main regulators of myocardial microcirculation, as well as aortic vasoreactivity. Furthermore, the decreased autonomic nervous system activity in SSc individuals may result in significant impairment of LV structure, function and mechanics [21]. Finally, as above mentioned, myocardial fibrosis could also play a prominent role [1]. In this regards, the imbalance between extracellular matrix synthesis and degradation by metalloproteinases has been highlighted as a prominent mechanism underlying impaired VAC.

Pulmonary arterial hypertension (PAH) typically affects the right ventricle, whereas the presence of LV abnormalities due to PAH is very uncommon (less than 1% of patients). In this regards, some studies have demonstrated the occurrence of right ventricular-arterial uncoupling in PAH but, to the best of our knowledge, no study have investigated or demonstrated the presence of high left VAC in PAH, due to the absence of a pathophysiological rationale. Moreover, the values of PAPs in our SSc patients with PAH are quite low (mean 34 mmHg), so the possibility of an impact on the left heart is highly unlikely. In line with this rationale, our study patients with VAC > 0.63 did not shown higher rate of PAH or higher level of pulmonary pressure values. Moreover, PAH was not a determinant of VAC in our linear regression analysis. Considering all this aspects, we did not consider useful to exclude these patients, which would considerably reduce the sample size of the study and its relevance.

VAC has been recently recognized as a key determinant of cardiovascular performance and its prognostic role has been demonstrated in various conditions [11]. For the first time, we provided data on the prognostic role of VAC in SSc, thus contributing to clarify the prognostic significance of subclinical cardiac alterations detected by imaging, one of the main unresolved issues in SSc. Our findings support a possible role for VAC in stratifying SSc patients with a major cardiovascular risk. Further prospective studies on larger cohorts are warranted to corroborate our findings.

While specific therapies for SSc cardiomyopathy are still lacking, vasoactive drugs have proven effective in mitigating myocardial perfusion and function abnormalities using conventional techniques. In addition, even low-dose acetylsalicylic acid has been recently associated with a lower incidence of distinct primary myocardial disease manifestations in SSc [22,23]. In this scenario, VAC evaluation may help identify patients who would most benefit from an early and more aggressive treatment with vasodilators and acetylsalicylic acid, to prevent myocardial dysfunction and reduce future MACEs. In this regards it is worth mentioning that—according to emerging evidences—even subclinical inflammation seems play a role in SSc cardiomyopathy. Given that systemic inflammation has been recognized as another potential pathogenetic mechanisms underlying VAC, its assessment might be useful in the longitudinal evaluation of SSc patients ad it pertains the potential benefit of immunosuppressants on subclinical myocardial dysfunction in SSc, as it has been suggested for rheumatoid arthritis.

As a limitation of the study, we should mention the relatively small sample size and monocentric nature of our study. Although statistically significant differences were observed, we acknowledge that our study may be slightly underpowered. A post-hoc power analysis (assuming α = 0.05) estimated that with 34 patients with VAC ≤ 0.63 and 31 patients with VAC > 0.63, with an event incidence of 44% in patients with VAC ≤ 0.63 and 74% in patients with VAC > 0.63, we reject the null hypothesis of equal survival with 75% power. In addition, we were not able to demonstrate the exact mechanisms underlying the subtle changes in myocardial contractility, based on other methods, such as cardiac magnetic resonance imaging. Myocardial fibrosis, which is a potential mechanism of myocardial dysfunction in SSc, was not investigated. Although we did not perform coronary angiography to exclude coronary heart disease, all patients were asymptomatic and the pre-test probability was low based on atherosclerotic risk factors, and there were no significant differences vs. controls. Moreover, we did not measure the global longitudinal strain (GLS) and therefore we do not have data of correlation between GLS and LV elastance. Therefore, because LGS is an early and well proved indicator of LV systolic dysfunction, it would be useful for identification of LV dysfunction in SSc patients.

## 4. Materials and Methods

### 4.1. Study Population

We conducted a retrospective cohort study that comprised patients attending the Rheumatology Unit of Padova University Hospital. The study population was retrieved from the database of our Echocardiography Laboratory. Overall, three hundred fifty patients underwent echocardiogram between January 2014 and March 2016 [24].

- Inclusion and Exclusion Criteria

**Figure 6:**
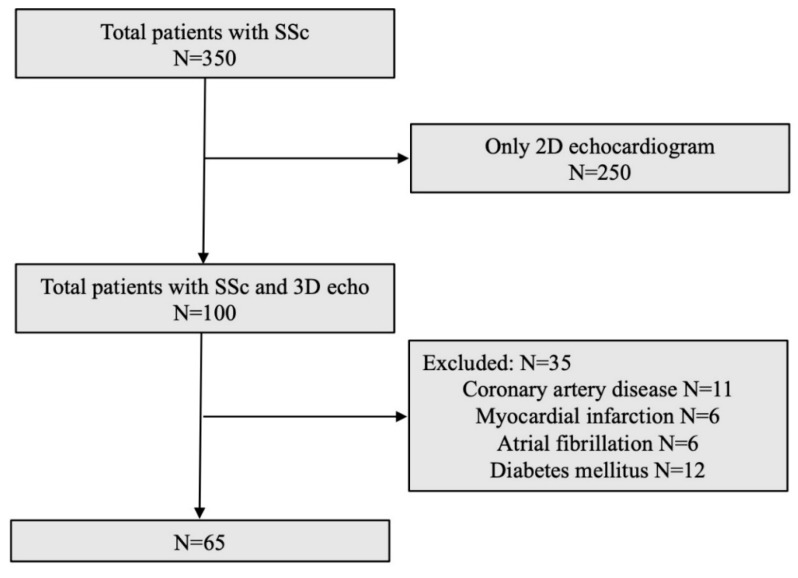
Study flow diagram. SSc, Sytemic Sclerosis.

Among the 350 patients, only those patients who were evaluated by 3DE were included (Figure 6). All patients were affected with SSc according to ACR/EULAR classification criteria [24].

Exclusion criteria were as follows: patients undergoing only 2DE (*n* = 250); patients (*n* = 35) with evidence of structural heart diseases (cardiomyopathy of any origin, significant valvular heart disease, coronary artery disease or myocardial infarction), atrial fibrillation, diabetes mellitus or systemic arterial hypertension grade II/III according to the European Society of Hypertension/European Society of Cardiology 2018 guidelines [25]; glomerular filtration rate <30 mL min^−1^ per 1.73 m^2^, cancer in the past 5 years, end-stage ILD and dyslipidemia.

Ultimately, we enrolled 65 SSc patients (54 female; age 56 ± 14 years) with no signs and symptoms of primary myocardial involvement (Figure 6), according to the available echocardiogram, and to clinical history, physical examination and ECG reported in clinical records within the previous six months.

Baseline evaluation included physical examination, gathering demographic and clinical data, and echocardiographic features (Table 1). Several disease features (e.g., cutaneous form, digital ulcers) and other organ involvement (e.g., interstitial lung disease, ILD) were recorded.

A series of 30 age- and sex-matched subjects satisfying the same exclusion criteria were evaluated by 3DE as controls. Given the retrospective nature of the study the written informed consent had been obtained by all patients at time of 3DE examination: this was a generic consensus to the acquisition of 3D images, beyond 2D standard Echocardiography.

### 4.2. Echocardiography

Echocardiography was performed using Vivid 7 ultrasound systems (GE Healthcare, Horten, Norway) with a 2.5-MHz transducer by 2 experienced cardiologists (F.T. and D.M.). All participants were examined with conventional 2-dimensional echocardiography and color tissue Doppler (TDI). All echocardiograms were stored on magneto-optical disks and an external FireWire hard drive (LaCie, France) and analyzed off line with commercially available software (EchoPac version 2008; GE Medical, Horten, Norway). Measurements of LV internal dimensions and LV mass index (LVMI) were performed and calculated according to European and American recommendations [26]. LV mass/body surface area ≤116 g/m^2^ in men and ≤104 g/m^2^ in women was considered normal. None of the patients suffered from significant valvular disease. In each subject, LVEF was measured and diastolic dysfunction was defined according to the American Society of Echocardiography criteria [27]. We considered abnormal an E/e′ > 14, and sign of diastolic dysfunction.

Echocardiographic parameters of diastolic function including the ratio between early (E) and late (A) peak velocities of the mitral inflow, E/A, and pulsed-wave tissue Doppler velocities of the mitral annulus in early diastole in the lateral wall (e′) were used as surrogates of LV diastolic relaxation and compliance and the deceleration time (DT) as a surrogate of early LV stiffness, and E/e′ as surrogate estimate of LV filling pressure [28]. All measures were averaged over 3 heart cycles.

#### 4.2.1. Transthoracic Real-Time 3D Imaging

Three-dimensional echocardiography data set acquisition of the LV was performed by the same examiner at the end of the standard 2DE examination using a 3Volume matrix-array transducer (GE Healthcare). A full-volume scan was acquired using second-harmonic imaging from apical approach, and care was taken to encompass the entire LV cavity in the data set. Consecutive four- to six-beat ECG-gated subvolumes were acquired during an end-expiratory apnoea to generate the full-volume data set. The quality of the acquisition was then verified in each patient by selecting twelve-slice display mode available on the machine to ensure that the entire LV cavity is included in the 3DE full volume, and, if unsatisfactory, the data set was re-acquired. Data sets were stored digitally in raw-data format and exported to a separate workstation equipped with commercially available software for offline analysis of LV volumes and LVEF from 3DE data sets: 4D AutoLVQ™ (EchoPac 202, GE Vingmed, Horten, Norway).

#### 4.2.2. Left Ventricular Volume Measurements

Left ventricular analysis was performed in several steps [29,30]:

(1) Automatic slicing of LV full-volume data set. The end-diastolic frames needed for contour detection were automatically displayed in quad-view: apical four-, two-chamber, long-axis views and LV short-axis plane. Each longitudinal view was color-coded and indicated on the short-axis image at 60° between each plane. Both reference frames in the end-systole and end-diastole could be also manually selected, if necessary.
(2) Alignment. Rapid manual alignment by pivoting and translating the four-chamber plane was first performed in order that the corresponding intersection line of all planes was placed in the middle of the LV cavity, crossing the LV apex and the center of mitral valve opening in each view. Aligning one plane automatically changed the others. Once LV central longitudinal axis was identified, accurate orientation of LV views was ensured by manual refinement of the angles between the LV planes on the LV short-axis view, in order to correspond to the defining anatomical landmarks of each view.
(3) Left ventricular reference point identification. To subsequently identify a fitting geometric model, the software required manual input of only two single points in any of the three LV apical planes (on points on mitral annulus, and one at the apex) first in end-diastolic frames, and then for corresponding end-systolic frames.
(4) Automated identification of endocardial border. The software automatically detected LV cavity endocardial border in 3D and provided the measured end-diastolic volume (LVEDV). Three additional short-axis views at different levels were displayed in order to facilitate verification of the accuracy of endocardial surface detection both in cross-section and in long-axis by rotating and translating active view plane. At this stage, LV borders could be manually adjusted, if unsatisfactory, by (dis)placing as many additional points as needed (manually corrected AutoLVQ), with secondary immediate automated refinement of boundary detection accordingly. This could be done on each of the six simultaneously displayed LV views, but also possible in between reference planes for LV with distorted shape. After completing steps 1–4 for end-diastolic views, only 3–4 sequence was required for end-systolic frames, since adjustments done in steps 1–2 were automatically carried out subsequently in end-systolic views.
(5) Final quantitative analysis and data display. Using the initial contours in both end-systole and end-diastole, a corresponding dynamic surface-rendered LV cast was derived. Final data panel automatically displayed LVEDV, LVESV, LVEF, SV, cardiac output, and heart rate values. A volume–time plot was also provided.

The intra- and inter-observer reproducibility for systolic function parameters in 20 randomly selected patients were good. Concordance between two raters using the Kappa statistic was 0.95 (*p* < 0.0001).

#### 4.2.3. Variables Derived from Left Ventricular Pressure-Volume Relations

**Figure 7:**
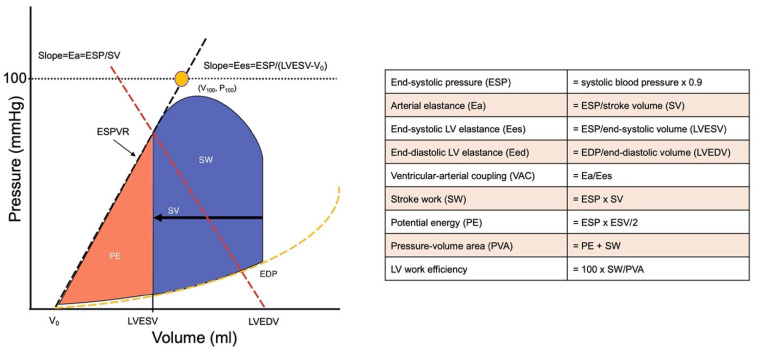
Pressure-volume loops of the left ventricle (**left**). Measurement of parameters derived from a pressure-volume loop of the left ventricle (**right**). End-systolic elastance (Ees) represents the slope of the end-systolic pressure volume relationship (ESPVR) where ESP denotes end-systolic pressure, and Ees represents the noninvasively derived single-beat estimation of this parameter. LVEDV is the end-diastolic volume, and LVESV is the end-systolic volume. V0 is the intercept of the ESPVR at an end-systolic pressure of 0 mm Hg, and V100 is the point on the end-systolic pressure volume line at an end-systolic pressure of 100 mm Hg. Effective arterial elastance (Ea) represents the negative slope joining the end-systolic pressure volume point to the point on the volume axis at end-diastole, where SV represents stroke volume.

To noninvasively quantify ventricular contractility, we calculated Ees as end-systolic pressure (ESP) divided by LVESV. LVEDV is an index of LV size and quantifies the degree of cardiac remodeling. The end-systolic pressure volume relationship (ESPVR) provides a load-independent measure of contractile function. The ESPVR is typically assumed to be linear and is therefore defined by a slope and an intercept. Although many studies focus on the slope alone, both the slope (end-systolic elastance [Ees]) and the intercept (V_0_) are required to describe the contractile state of the left ventricle. Ees quantifies ventricular elastance (stiffness) at end-systole, and V_0_ is a measure of ventricular volume at a theoretical end systolic pressure of 0 mm Hg. Because V_0_ is an extrapolated value obtained at a non-physiological pressure, the LVESV at a systolic pressure of 100 mm Hg (V_100_) is also often described. For arterial load, Ea was the ratio of ESP to stroke volume (SV), and VAC was defined as the ratio of Ea to Ees. For these equations, LVESV and SV were obtained from 3DE results. ESP was defined as 0.9 x systolic blood pressure determined by noninvasive blood pressure measurement at the same time as 3DE. As recommended by the ESC guidelines on hypertension, patients were seated comfortably in a quiet environment for 5 min before beginning blood pressure measurements. Three blood pressure measurements were recorded, 1–2 min apart, and additional measurements only if the first two readings differed by >10 mmHg. We used a standard bladder cuff (12–13 cm wide and 35 cm long) for all patients and controls. End-diastolic elastance (Eed) was the ratio of left ventricular end-diastolic pressure (EDP) to LVEDV. We estimated EDP with a formula using the E/e’ ratio (11.96 + 0.596 E/e’) [31].We estimated mechanical energy including SW, PE, PVA, and LV mechanical efficiency [32]. (Figure 7).

### 4.3. Primary Study Endpoint: Major Adverse Cardiovascular Events (MACEs) during Follow-up

The primary study endpoint was a composite endpoint of MACEs during follow-up. MACEs were defined by the occurrence of death for heart failure or hospitalization from CV causes (i.e., angina, myocardial infarction or heart failure). Angina and myocardial infarctions were defined according to ESC guidelines [33,34]. Two physicians (E.Z. and E.B.) blinded to 3DE findings reviewed all the medical records of included patients, regularly follow-up every 6 months—as per usual protocol at our Rheumatology Unit. In addition, further information were also obtained by evaluating hospital discharge cards and the personal status (i.e., alive/dead) that is recorded in the medical information system of our region.

### 4.4. Statistical Analysis

Continuous variables with no/mild skew were presented as mean ± SD; skewed measures were represented as median with first and third quartiles (Q1-Q3). Discrete variables were summarized as frequencies and percentages. The distribution of the data was analysed with a 1-sample Kolmogorov-Smirnov test. Categorical variables were compared by the χ2 test or the Fisher exact test as appropriate. Continuous data were compared using the 2-tailed unpaired t test (for normally distributed data sets) or the Mann-Whitney U test (for skewed variables). Time-dependent receiver operating characteristic curves were used to determine the optimal cutoffs for the primary composite endpoint based on the Youden index. Bivariate correlations were assessed by the Spearman coefficient (ρ). In unadjusted and multivariable-adjusted linear regression analyses, we expressed association between VAC and other clinical variables. Logistic regressions with odds ratios (ORs) and 95% confidence intervals (CIs) were applied to investigate associations between VAC > 0.63 and clinical characteristics. Event rates are plotted in Kaplan-Meier curves for the primary composite end point and cardiovascular death, and groups were compared using the log-rank test. Univariate and multivariable Cox proportional hazards models were performed to identify the independent determinants of the primary composite end point. Variables with *p* < 0.05 at univariate analysis were included as covariables in multivariable models. Multivariable analyses were performed using a backward-conditional selection procedure on the remaining variables demonstrated a *p* value < 0.05. Pulmonary hypertension, dcSSc and interstitial lung disease, which have proven important in systemic sclerosis, were forced into the multivariable models, because model’s adjustments should take into account factors with well-established clinical relevance. Moreover, VAC was introduced separately in the multivariable analysis to compare incremental value in predicting outcome. To assess the incremental value of VAC in addition to other risk factors for predicting adverse events, we calculated the improvement in global χ^2^ value. Multivariable Cox models were discriminated by the C-index (values > 0.7 were deemed acceptable). The agreement between 2D-or 3D-echocardiography was tested by the Bland-Altman method and by the concordance correlation coefficient comparing the mean differences between the two methods of measurements and 95% limits of agreement as the mean difference. Intraobserver and interobserver reproducibilities of VAC were evaluated by linear regression analysis and expressed as correlation of coefficients (*r*) and standard error of estimates (SEE), and by the intraclass correlation coefficient. Reproducibility is considered satisfactory if the intraclass correlation coefficient is between 0.81 and 1.0. Intraobserver and interobserver reproducibility measurements were calculated in all 65 patients. All tests were two-sided and statistical significance was accepted if the null hypothesis could be rejected at *p* < 0.05. Data were analyzed with SPSS software version 24.0 (SPSS, Inc., Chicago, IL, USA). The study was approved by the institutional ethics committee.

## 5. Conclusions

In conclusion, our results may help better identify primary cardiac involvement in SSc. We provided the first evidence that VAC may be impaired in SSc and, importantly, that it seems to play a prognostic role in these patients. Our results also suggest that patients with dcSSc present an intrinsic LV systolic dysfunction, which seems to worsen over time and is responsible for the LV inability to compensate higher afterload. Further prospective studies are warranted to ascertain whether early intervention can improve outcomes in patients with “abnormal” VAC.

## Figures and Tables

**Table 1:** Clinical and Echocardiographic Features in SSc Patients with and without VAC > 0.63.

	**All Patients** **(n = 65)**	**VAC ≤ 0.63** **(n = 34)**	**VAC > 0.63** **(n = 31)**	***p* Value**
Age, years	56 ± 14	58 ± 13	53 ± 16	0.12
Female, n (%)	54 (83)	29 (85)	25 (81)	0.61
Body weight, Kg	60 ± 11	62 ± 9	59 ± 10	0.81
BMI, Kg/m^2^	25 ± 2	26 ± 3	25 ± 1	0.80
Systolic blood pressure, mmHg	126 ± 21	127 ± 20	125 ± 22	0.55
Diastolic blood pressure, mmHg	74 ± 10	70 ± 9	79 ± 6	0.69
Hemoglobin, g/dL	14 ± 0.8	15 ± 0.6	13 ± 0.2	0.71
Creatinine, mg/dL	0.98 ± 0.02	0.95 ± 0.01	1.02 ± 0.02	0.81
**Clinical Features**				
Disease duration, years	19 ± 11	16 ± 9	22 ± 12	0.03
Diffuse cutaneous form, n (%)	27 (41)	10 (29)	17 (55)	0.03
PAH, n (%)	22 (34)	6 (17)	16 (47)	0.57
ILD on HRCT, n (%)	37 (57)	20 (59)	17 (55)	0.86
Digital ulcers, n (%)	39 (60)	20 (58)	19 (61)	0.66
**Treatment, n (%)**				
Prostanoid ev	12 (18)	4 (12)	8 (26)	0.40
ET-1 inhibitors	22 (34)	14 (41)	8 (26)	0.33
Immunosuppressants	30 (46)	18 (53)	12 (39)	0.28
**Echocardiographic Measurements**				
LVEDD, mm	44.9 ± 0.5	45.1 ± 0.6	44.7 ± 0.5	0.94
IVS thickness, mm	11.4 ± 1.2	9.9 ± 1.9	13.6 ± 1.6	0.28
PW thickness, mm	11.5 ± 1.3	9.6 ± 1.9	13.2 ± 1.6	0.21
LV mass, g	155 ± 59	151 ± 63	161 ± 53	0.33
LVEDV, mL	88 ± 26	85 ± 29	91 ± 24	0.24
LVESV, mL	33 ± 11	28 ± 10	38 ± 11	<0.0001
SV, mL	55 ± 17	57 ± 19	52 ± 14	0.41
LVEF (%)	62 ± 5	67 ± 3	57 ± 2	<0.0001
Aorta, mm	30 ± 0.3	30 ± 0.3	30 ± 0.4	0.69
Left atrium, mm	49.8 ± 9	47.7 ± 8	51.9 ± 9	0.12
RVEDD, cm^2^	19 ± 5	18.2 ± 5	20 ± 5	0.08
TAPSE, cm	2.25 ± 0.5	2.31 ± 0.5	2.19 ± 0.5	0.24
Peak E velocity, m/s	0.86 ± 0.2	0.85 ± 0.1	0.88 ± 0.2	0.58
Peak A velocity, m/s	0.79 ± 0.2	0.84 ± 0.2	0.75 ± 0.2	0.13
DT, ms	199 ± 60	219 ± 62	179 ± 52	0.08
E/A ratio	1.14 ± 0.3	1.07 ± 0.3	1.21 ± 0.3	0.16
E/e’ ratio	10.2 ± 4.3	10.9 ± 4.7	9.3 ± 3.6	0.23
PAP, mmHg	34 ± 19	32 ± 16	37 ± 21	0.42
**Pressure-Volume Curve Relationships**				
End-diastolic elastance, mmHg/mL	0.21 (0.17–0.28)	0.23 (0.17–0.29)	0.21 (0.16–0.24)	0.42
Arterial elastance, mmHg/mL	2.10 (1.82–2.80)	2.10 (1.78–3.01)	2.10 (1.87–2.80)	0.69
End-systolic elastance, mmHg/mL	3.79 (2.87–5.30)	4.43 (3.3–6.3)	2.94 (2.53–4.12)	<0.0001
Ventricular-arterial coupling	0.57 (0.49–0.72)	0.51 (0.45–0.53)	0.73 (0.68–0.75)	<0.0001
Stroke work, mmHg·mL	6021 (4275–8424)	6351 (4325–8991)	5346 (4252–7695)	0.38
Potential energy, mmHg·mL	1566 (1258–2413)	1532 (1194–2103)	1935 (1521–3087)	0.001
Pressure-volume area, mmHg·mL	7659 (5798–11,102)	8008 (5550–11,274)	7281 (5798–10,410)	0.86
LV efficiency, %	78 (73–80)	79 (78–81)	73 (72–74)	<0.0001

BMI, body mass index; DT, E-wave deceleration time; ET-1, endothelin 1; E/A, ratio of early transmitral diastolic flow velocity (E) and flow velocity during atrial contraction (A); HRCT, high resolution computed tomography; ILD, interstitial lung disease; IVS, interventricular septum; LV, left ventricle; LVEDD, left ventricular end-diastolic diameter; LVEDV, left ventricular end-diastolic volume; LVEF, left ventricular ejection fraction; LVESV, left ventricular end-systolic volume; PAH, pulmonary arterial hypertension; PAP, pulmonary arterial pressure; PW, posterior wall; RP, Raynaud phenomenon; RVEDD, right ventricular end-diastolic dimension; SSc, systemic sclerosis; SV, stroke volume; TAPSE, tricuspid annular plane excursion. Values are mean ± SD or median (IQR).

**Table 2:** Clinical and Echocardiographic Features in dcSSc Patients versus lcSSc Patients.

	**dcSSc** **(n = 27)**	**lcSSc** **(n = 38)**	***p* Value**
Age, years	51 ± 14	59 ± 14	0.02
Female, n (%)	19 (70)	35 (92)	0.01
Body weight, Kg	60 ± 5	57 ± 2	0.52
BMI, Kg/m^2^	26 ± 2	25 ± 2	0.61
Systolic blood pressure, mmHg	124 ± 22	128 ± 21	0.44
Diastolic blood pressure, mmHg	77 ± 8	72 ± 7	0.62
Hemoglobin, g/dL	13 ± 0.3	15 ± 0.1	0.58
Creatinine, mg/dL	0.98 ± 0.01	0.93 ± 0.03	0.34
Clinical features			
Disease duration, years	14 ± 7	22 ± 12	0.005
PAH, n (%)	10 (37)	12 (31)	0.25
ILD on HRCT, n (%)	12(44)	25 (66)	0.01
Digital ulcers, n (%)	17 (62)	22 (58)	0.83
**Treatment, n (%)**			
Prostanoid ev	7 (26)	5 (13)	0.21
ET-1 inibithors	9 (33)	13 (34)	0.44
Immunosuppressants	8 (29)	22 (58)	0.008
**Echocardiographic measurements**			
LVEDD, mm	46 ± 0.6	44 ± 0.5	0.07
IVS thickness, mm	10 ± 0.2	12 ± 0.2	0.46
PW thickness, mm	10 ± 0.2	12 ± 0.3	0.83
LV mass, g	148 ± 51	166 ± 71	0.54
LVEDV, mL	101 ± 29	82 ± 21	0.004
LVESV, mL	39 ± 12	29 ± 9	0.001
SV, mL	62 ± 20	52 ± 13	0.03
LVEF (%)	60 ± 6	64 ± 5	0.01
Aorta, mm	29 ± 0.4	30 ± 0.3	0.12
Left atrium, mm	48 ± 0.8	50 ± 0.9	0.50
RVEDD, cm^2^	19.9 ± 5	18.3 ± 5	0.32
TAPSE, cm	2.24 ± 0.5	2.30 ± 0.5	0.65
Peak E velocity, cm/s	0.90 ± 0.2	0.84 ± 0.2	0.53
Peak A velocity, cm/s	0.81 ± 0.2	0.78 ± 0.2	0.53
DT, ms	180 ± 54	211 ± 61	0.38
E/A ratio	1.19 ± 0.3	1.11 ± 0.2	0.44
E/e’ ratio	8.6 ± 2	10.7 ± 4	0.07
PAP, mmHg	32 ± 3	34 ± 3	0.75
**Pressure-volume curve relationships**			
End-diastolic elastance, mmHg/mL	0.17 (0.13–0.22)	0.23 (0.19–0.28)	0.03
Arterial elastance, mmHg/mL	1.83 (1.53–2.20)	2.21 (1.88–2.73)	0.01
End-systolic elastance, mmHg/mL	2.90 (2.22–3.56)	4.06 (3.12–5.49)	0.001
Ventricular-arterial coupling	0.69 (0.52–0.74)	0.52 (0.45–0.65)	0.02
Stroke work, mmHg·mL	6284 (4045–8748)	5805 (4680–8748	0.25
Potential energy, mmHg·mL	1863 (1493–2973)	1552 (1215–2268	0.01
Pressure-volume area, mmHg·mL	8008 (5487–11,522)	7357 (6138–11,016	0.12
LV efficiency, %	74 (72–78)	79 (76–81)	0.02

Abbreviations as in Table 1. Values are mean ± SD or median (IQR).

**Table 3:** Independent Effects of Clinical Variables on VAC.

	b	95% CI	*p* Value
dcSSc	0.342	0.020–0.184	0.01
Prostanoid ev	0.247	(−0.008)–0.154	0.07
Disease duration	0.133	(−0.002)–0.005	0.39
Age at SSc diagnosis	−0.077	(−0.004)–0.002	0.63
Corrected R^2^			0.008

Note: Using multivariable linear regression analysis with stepwise method.

**Table 4:** Clinical and Echocardiographic Features in Patients with and without MACEs.

	**No MACEs** **(n = 27)**	**MACEs** **(n = 38)**	***p* Value**
Age, years	55 ± 11	56 ± 16	0.66
Female, n (%)	22 (81)	32 (84)	0.77
Body weight, Kg	58 ± 3	59 ± 2	0.81
BMI, Kg/m^2^	25 ± 1	26 ± 2	0.89
Systolic blood pressure, mmHg	133 ± 19	122 ± 22	0.04
Diastolic blood pressure, mmHg	75 ± 6	73 ± 7	0.49
Hemoglobin, g/dL	14 ± 0.3	15 ± 0.4	0.68
Creatinine, mg/dL	0.95 ± 0.04	0.96 ± 0.01	0.89
**Clinical features**			
Disease duration, years	10 ± 1	16 ± 1	0.03
dcSSc, n (%)	9 (33)	16 (42)	0.43
PAH, n (%)	8 (30)	14 (36)	0.69
ILD on HRCT, n (%)	10 (37)	27 (71)	0.03
Digital ulcers, n (%)	16 (59)	23 (60)	0.75
**Treatment, n (%)**			
Prostanoid ev	6 (22)	6 (15)	0.64
ET-1 inibithors	9 (33)	13 (34)	0.44
Immunosuppressants	6 (22)	24 (63)	0.007
**Echocardiographic measurements**			
LVEDD, mm	43 ± 0.5	45 ± 0.5	0.13
IVS thickness, mm	10 ± 0.2	13 ± 0.2	0.18
PW thickness, mm	9 ± 0.2	12 ± 0.3	0.22
LV mass, g	143 ± 50	168 ± 66	0.17
LVEDV, mL	85 ± 24	91 ± 28	0.36
LVESV, mL	30 ± 10	35 ± 12	0.06
SV, mL	54 ± 15	55 ± 18	0.84
LVEF (%)	64 ± 5	60 ± 5	0.01
Aorta, mm	30 ± 0.3	29 ± 0.3	0.46
Left atrium, mm	52 ± 0.9	48 ± 0.8	0.13
RVEDD, cm^2^	17.7 ± 3	20 ± 6	0.12
TAPSE, cm	2.35 ± 0.5	2.15 ± 0.5	0.23
Peak E velocity, cm/s	0.92 ± 0.2	0.82 ± 0.3	0.50
Peak A velocity, cm/s	0.80 ± 0.2	0.73 ± 0.2	0.41
DT, ms	189 ± 50	200 ± 55	0.36
E/A ratio	1.17 ± 0.3	1.13 ± 0.2	0.71
E/e’ ratio	11 ± 4	9.2 ± 4	0.24
PAPs, mmHg	29 ± 9	37 ± 23	0.14
**Pressure-volume curve relationships**			
End-diastolic elastance, mmHg/mL	0.23 (0.18–0.27)	0.19 (0.16–0.24)	0.68
Arterial elastance, mmHg/mL	1.25 (1.96–2.84)	1.95 (1.69–2.45)	0.52
End-systolic elastance, mmHg/mL	4.50 (3.08–6.08)	3.30 (2.70–3.89)	0.01
Ventricular-arterial coupling	0.51 (0.45–0.64)	0.63 (0.53–0.72)	0.008
Stroke work, mmHg·mL	6588 (4781–8910)	5400 (4230–6705)	0.44
Potential energy, mmHg·mL	1521 (1257–2322)	1748 (1527–2252)	0.52
Pressure-volume area, mmHg·mL	8100 (6169–11,381)	7380 (5620–9459)	0.67
LV efficiency, %	79 (75–81)	75 (73–78)	0.01

Abbreviations as in Table 1. Values are mean ± SD or median (IQR).

**Table 5:** Univariate and Multivariable Predictors of MACEs.

	**Univariate**		**Multivariable Model**	
	**HR (95% CI)**	***p* Value**	**HR (95% CI)**	***p* Value**
Age > 60 years	1.5 (1.2–2.9)	0.20		
Female	2.0 (1.2–5.2)	0.15		
Disease duration, years	1.03 (1.006–1.06)	0.01		
Diffuse cutaneous form	1.7 (1.1–3.3)	0.13		
PAH	1.0 (0.3–3.4)	0.94		
ILD on HRCT	1.2 (0.5–2.6)	0.54		
Immunosuppressants	1.7 (0.8–3.8)	0.13		
**Echocardiographic measurements**				
LVEDV > 85 mL	1.0 (0.5–1.9)	0.97		
LVESV > 34 mL	1.1 (0.6–2.2)	0.61		
SV < 53 mL	1.3 (0.7–2.6)	0.31		
LVEF < 62%	2.1 (1.0–4.1)	0.02		
TAPSE < 2.1 cm	1.6 (0.6–4.3)	0.29		
E/e’ ratio > 9	2.1 (0.6–6.8)	0.21		
PAP > 30 mmHg	0.8 (0.4–1.6)	0.56		
**Pressure-volume curve relationships**				
End-diastolic elastance > 0.21 mmHg/mL	1.0 (0.3–3.2)	0.86		
Arterial elastance > 2 mmHg/mL	0.8 (0.4–1.5)	0.56		
End-systolic elastance < 3.4 mmHg/mL	1.4 (0.7–2.8)	0.24		
Ventricular-arterial coupling > 0.63	2.4 (1.2–4.8)	0.008	2.5 (1.13–5.7)	0.01
Stroke work < 5671 mmHg·mL	1.5 (0.8–2.9)	0.19		
Potential energy > 1621 mmHg·mL	1.1 (0.6–2.2)	0.62		
Pressure-volume area < 7498 mmHg·mL	0.9 (0.4–1.7)	0.75		
LV efficiency < 76%	2.1 (1.09–4.1)	0.02		

CI. confidence interval; HR, hazard ratio; HRCT, high resolution computed tomography; ILD, interstitial lung disease; LVEDV, left ventricular end-diastolic volume; LVEF, left ventricular ejection fraction; LVESV, left ventricular end-systolic volume; MACE, major adverse cardiac events; PAH, pulmonary arterial hypertension; PAP, pulmonary arterial pressure; SV, stroke volume; TAPSE, tricuspid annular plane excursion.

**Table 6:** Arterial elastance, End-systolic elastance and Ventricular-arterial coupling by 2D and 3D echocardiography (*n* = 65).

	2D Echo	3D Echo	*p* Value
Arterial elastance, mmHg/mL	2.29 (1.93–2.70)	2.04 (1.77–2.66)	0.23
End-systolic elastance, mmHg/mL	4.17 (3.38–4.97)	3.41 (2.57–4.50)	0.02
Ventricular-arterial coupling	0.57 (0.44–0.66)	0.62 (0.48–0.71)	0.15

## Data Availability

The data sets used and/or analyzed during the current study are available from the corresponding author on reasonable request.
